# Estimation of minimally important differences in the EQ-5D and SF-6D indices and their utility in stroke

**DOI:** 10.1186/s12955-015-0227-3

**Published:** 2015-03-09

**Authors:** Sang-Kyu Kim, Seon-Ha Kim, Min-Woo Jo, Sang-il Lee

**Affiliations:** Department of Preventive Medicine, Dongguk University College of Medicine, 123, Dongdae-ro, Gyeongju-si, Gyeongbuk, South Korea; Department of Nursing, Dankook University, 119 Dandae-ro, Dongnam-gu, Cheonan-si, Chungnam South Korea; Department of Preventive Medicine, University of Ulsan College of Medicine, 88, Olympic-ro 43-gil, Songpa-gu, Seoul 138-736 South Korea

**Keywords:** EQ-5D, SF-6D, Health-related quality of life, Minimally important difference, Stroke

## Abstract

**Background:**

The aim of the present study was to estimate minimally important differences (MIDs) in EQ-5D and SF-6D indices and to explore the responsiveness of EQ-5D and SF-6D indices in stroke.

**Methods:**

We used observational longitudinal survey data of EQ-5D and SF-36 that were administered to stroke patients at baseline and at 10 months. A range of MIDs for both indexes was estimated using anchor-based approaches. The modified Rankin scale and the Barthel index were used as an anchor.

**Results:**

The MID estimates for EQ-5D ranged from 0.08 to 0.12 and those for SF-6D ranged from 0.04 to 0.14 in stroke patients. The MID values for these two utility measures differed in absolute magnitude, as the SF-6D index has wider range that that of the EQ-5D index.

**Conclusions:**

The MID values for these two utility measures differed in absolute magnitude, as the SF-6D index has wider range that that of the EQ-5D index. These MID estimates may assist the interpretation of health related quality of life assessments related to health care intervention in stroke patients.

## Background

In 2010, the mortality rate due to stroke was 53.2 per population of 100,000 in Korea, making it the most common single disease responsible for death in Korea [1]. Given that stroke is a major cause of disability, the quality of life following stroke can be as imperative as the duration of life after stroke. Health-related quality of life (HRQoL) is a person’s actual or expected physical, emotional and social well-being resulting from a medical condition or its treatment [2]. The use of HRQoL instruments has become increasingly common in stroke assessments [3,4].

Although HRQoL is currently recognized as an important endpoint in clinical trials, the meaningfulness of HRQoL scores may not be apparent to patients, clinicians or researchers [5]. Schünemann & Guyatt have stipulated that a minimally important difference (MID) of any HRQoL measure as the “smallest difference in score in the outcome of interest that informed patients or informed proxies perceive as important, either beneficial or harmful, and which would lead the patient or clinician to consider a change in the management” [6]. Interpretation of scores is an important issue in the field of HRQoL measurement, but there is no consensus regarding the most appropriate method for assessing the ability of an instrument to capture meaningful differences [7]. The MIDs has been determined using both, anchor-based and distribution-based methods. The anchor-based method was an approach to find out HRQoL score changes on minimal changes in clinical measures, which are defined as anchors, across multiple time points. Clinical measures can be objective indicators or subjective assessments of a patient status [8]. Estimating the MID is a special case of examining responsiveness to change [9] Responsiveness has been defined as the ability to detect changes that are meaningful or clinically important [10]. When a HRQoL instrument is more responsive, it has the advantage of requiring smaller sample sizes to demonstrate clinically important effects [11] and is able to capture changes when those meaningfully occur. Distribution-based methods makes it possible to compare change observed for measures that have a different raw metric, and the degree of deviation within the sample without reference to an external standard and provide no direct information about the MID [9,12]. The role of the distribution-based approaches is identifying the minimum detectable change (MDC), and the MDC cannot universally and reliably replace the MID [13,14]. Therefore, anchor-based measures are the only way to estimate the MID directly [9,13,14].

The EQ-5D [15] is a generic preference-based HRQoL instrument that generates utility scores that are used for economic evaluation in the calculation of quality-adjusted life years (QALYs). A systematic review concluded that the EQ-5D was the most frequently used questionnaire in cost–utility studies including QALYs [16]. Worldwide, the short form-36 version 2 (SF-36 v2) is one of the most popular generic instrument to measure HRQoL beyond cost-utility studies. The SF-36 v2 is a short-form health survey with 36 questions that yields an eight-scale profile (PF, physical functioning; RP, role physical; BP, bodily pain; GH, general health; VT, vitality; SF, social functioning; RE, role emotional; MH, mental health) of functional health and well-being, as well as two psychometrically based summary measures of physical and mental health and a preference-based health utility index [17].

The MIDs for the EQ-5D or SF-6D index in patients with various diseases have been investigated [18-20], although the value of these studies is limited by the small sample sizes. To our knowledge, none of the studies that have considered the responsiveness of HRQoL instruments in community-based stroke patient field have estimated MIDs. The purpose of this article was to estimate MIDs for the EQ-5D and SF-6D indices and to explore the responsiveness for the EQ-5D and SF-6D indices in patients with stroke.

## Methods

### Subjects

Subjects who had suffered first ever or recurrent stroke and were aged 50 years or older were invited to participate in our research using the registry of disabled persons in Gyeong-ju city. Subjects disabled due to brain tumor, Parkinson disease or brain trauma were excluded from the study. Interviewers were nurses working in the community health center. Those nurses were trained for 4 hours before survey and then they visited the subject residences (home or nursing home). The first survey was performed from July 2008 until October 2008 and the second survey was conducted from May 2009 until July 2009. Both surveys used the same questionnaire including demographic factors, clinical information, and quality of life information such as EQ-5D, and SF-36 v2. This study was approved by the Institutional Review Board of Dongguk University, Gyeongju Hospital (approval number: DUGH 10–35). All participants provided written informed consent.

### Measures

General and clinical characteristics, the Modified Rankin Scale (MRS) and the Barthel ADL index (BI) were gathered by trained interviewers who were registered nurses. The EQ-5D and the SF-36 v2 were self-administered with or without assistant. The EQ-5D is a generic preference-based measure that health status describes in terms of five dimensions: mobility, self-care, usual activities, pain discomfort and anxiety/depression. Each dimension has three levels, indicating no problems, some or moderate problems and extreme problems [15]. The EQ-5D index of health state was calculated using the valuation set of the Korean population [21]. Therefore, the possible range of EQ-5D scores was from −0.171 to 1.0, with 1.0 denoting full health (11111 state), and 0.0 denoting as bad as being dead. The SF-6D utility score could be calculated using Brazier’s et al’s algorithm, which was recommended by authors (model 10) [22]. The SF-6D consists of six dimensions (i.e., physical functioning, role limitations, social functioning, pain, mental health and vitality) and each dimension can be ranked in terms of between four and six levels. The SF-6D index was elicited from a preference-based algorithm, which was developed by the standard gamble method for the population of the United Kingdom [22] because a Korean valuation set for SF-6D was not available. Therefore, the possible range of the SF-6D is from 0.296–1.0.

The MRS is a measure of disability. The scale consists of six grades from 0–5, with 0 corresponding to no symptoms and 5 corresponding to severe disability [23]. The BI is a measure of the ability to perform basic activities associated with daily living. We used the Korean version of BI [24]. This is based on Collin’s modified BI, which ranges from 0–20 [25]. A higher BI score indicates more independence in physical functioning. There was evidence on validity and reliability of EQ-5D, SF-36 and BI in Korean population [26-29].

### Analyses

We assessed the usefulness of anchors by investigating the correlation between the changes of index scores and the anchor-change score. Yost and Eton suggested that the anchor change scores and HRQoL change scores should be linearly related and have at least a moderate correlation [5]. We used the change of MRS and BI to determine anchor-based differences because it fulfilled this suggestion (Pearson correlation coefficients between anchor change scores and HRQoL change scores ranged from 0.46 to 0.55). The change of MRS was arbitrarily classified based on the movement between grades at baseline and 10 months on the MRS: no change (no movement), minimally better (improvement of l grade), sizeable better (improvement of more than l level), minimally worse (deterioration of l grade), and sizeable worse (deterioration of more than l level). As suggested in previous publications, we considered a difference of at least four points as a significant difference of scores in case of BI [25]. Groups were classified based on the range of changes in the BI score. Those classifications were either: no change (change from −3 to 3 points), minimally better (increase from 4 to 6 points), sizable better (increase of more than 6 points), minimally worse (decrease from 4 to 6 points), and sizable worse (decrease of more than 6 points).

We considered the mean score difference of both indices in ‘minimally better’ and ‘minimally worse’ categories to be an adequate estimate of MID. The significance of differences in mean score changes between any two time points was tested using the Wilcoxon signed-rank test.

Responsiveness of both of the EQ-5D and the SF-6D indices were compared using effect size (ES) and the standardized response mean (SRM). ES was calculated as the ratio between the mean change scores and the standard deviation of baseline scores [30]. The SRM was calculated as a ratio of mean change scores to the SD of the change scores [31]. Both, effect size and SRM were interpreted using benchmarks for effect size. Whereas 0.2 was interpreted as a small magnitude of effect, 0.5 indicated a medium effect and 0.8 was interpreted as a large effect [32].

We excluded subjects who missed any items in either EQ-5D or SF-6D. Subjects were additionally excluded in either MRS-based or BI-based analysis if the MRS or BI index were not completed at either time point, therefore there were two different analysis sets.

## Results

### General characteristics

Of 991 potential subjects, 541 persons participated in both interviews. Of the participants, 54 were excluded from the analysis owing to missing answers for either EQ-5D or SF-6D items in the SF-36 questionnaire. Thus, the final analysis set consisted of 487 subjects. The mean age of the subjects was 68.3 years (SD 8.1) and 58.9% were men. Regarding MRS scores for subjects at baseline, 1% had no symptoms, 11.5% had no significant symptoms, 31.1% presented with slight disability, 32.5% presented with moderate disability, 15.6% presented with moderately severe disability and 7.8% presented with severe disability (Table 1). First ever stroke counted for 332 (69.8%) and years since first stroke occurred counted for 9.0 (SD 7.3).

**Table 1:** **General characteristics of study subjects at baseline (n = 487)**

**Variables**	**N**	**(%)**
Age, years		
50-59	77	(15.8)
60-69	184	(37.8)
70-79	186	(38.2)
80 and more	40	(8.2)
Mean (SD)	68.3	(8.1)
Gender		
Male	287	(58.9)
Female	200	(41.1)
Proportion of reporting problems in EQ-5D		
Mobility	430	(88.3)
Self-care	387	(79.5)
Usual activities	436	(89.5)
Pain/discomfort	410	(84.2)
Anxiety/depression	351	(72.1)
Modified Rankin Scale		
0	5	(1.0)
1	56	(11.5)
2	151	(31.1)
3	158	(32.5)
4	76	(15.6)
5	38	(7.8)
Barthel ADL index		
Mean (SD)	14.8	(6.0)

### Anchor-based approach

The mean changes, SRM and ES in the EQ-5D and SF-6D indices according to categories of change in MRS and BI are listed in Table 2. Most subjects who improved or deteriorated showed significant changes in the scores of both, the EQ-5D and SF-6D indices. for the EQ-5D using MRS as the basis for the anchor-based method ranged from 0.08 (as seen in the minimal improvement group to 0.12 (as seen in the minimal deterioration group) The estimated MID for the SF-6D index using the MRS as the basis for the anchor-based method ranged from 0.04 (as seen in the minimal deterioration group) to 0.07 (as seen in the minimal improvement group). The estimated MID using the BI as the basis for the anchor-based method ranged from 0.09 at minimal improvement to 0.12 at minimal deterioration for the EQ-5D index, whereas it ranged from 0.04 at minimal deterioration to 0.14 at minimal improvement for the SF-6D index. The magnitude and pattern in SRM and ES for BI-anchored responsiveness were similar with MRS-anchored responsiveness. The SRM and ES for both, the EQ-5D index and SF-6D index were of similar magnitude and pattern using the MRS and BI anchored approaches. For patients in the sizable better and minimally better categories, the EQ-5D was less responsive than SF-6D, whereas for patients classified as being either sizable worse or minimally worse, the EQ-5D was more responsive than SF-6D.

**Table 2:** **Anchor-based approach for determining minimally important differences for the EQ-5D and SF-6D indices**

	**Modified rankin Scale based**						**Barthel ADL index based**					
	**N**	**Mean change (SD) (time2-time1)**		**P-value** ^**†**^	**SRM**	**ES**	**N**	**Mean change (SD) (time2-time1)**		**P-value** ^**†**^	**SRM**	**ES**
EQ-5D index												
All	484	−0.04	(0.27)	0.003	0.15	0.07	442	−0.05	(0.26)	0.001	0.17	0.08
Sizeable better	17	0.17	(0.26)	0.015	0.66	0.71	23	0.23	(0.31)	<0.001	0.76	0.72
Minimally better	86	0.08*	(0.21)	<0.001	0.38	0.28	27	0.09*	(0.28)	0.048	0.32	0.31
No change	220	−0.01	(0.22)	0.413	0.06	0.03	281	−0.01	(0.19)	0.471	0.06	0.03
Minimally worse	115	−0.12*	(0.26)	<0.001	0.47	0.46	53	−0.12*	(0.23)	<0.001	0.50	0.43
Sizeable worse	46	−0.27	(0.26)	<0.001	0.75	0.84	58	−0.33	(0.34)	<0.001	0.97	1.27
P-value^§^		<0.001						<0.001				
SF-6D index												
All	484	0.01	(0.14)	0.520	0.04	0.01	442	0.01	0.14	0.334	0.05	0.01
Sizeable better	19	0.16	(0.13)	0.002	1.21	1.33	23	0.14	(0.12)	<0.001	1.17	1.40
Minimally better	86	0.07*	(0.13)	<0.001	0.52	0.58	27	0.14*	(0.15)	<0.001	0.92	1.11
No change	220	0.01	(0.13)	0.296	0.07	0.08	281	0.01	(0.14)	0.373	0.05	0.08
Minimally worse	115	−0.04*	(0.12)	<0.001	0.33	0.36	53	−0.04*	(0.13)	0.068	0.27	0.33
Sizeable worse	46	−0.08	(0.12)	<0.001	0.50	0.53	58	−0.06	(0.12)	<.0001	0.53	0.55
P-value^§^		<0.001					<0.001					

SRM = standardized response mean; ES = effect size.

*These scores are estimates of the MID.

^†^P-value by Wilcoxon signed rank test.

^§^P-value by Kruskal Wallis test.

## Discussion

This study estimated MIDs using anchor -based methods in stroke patients of a community-based cohort. This study showed that the MID estimates for EQ-5D ranged from 0.08–0.12 whereas the MID estimates for SF-6D ranged from 0.04–0.14. The distribution- and anchor-based estimates tended to converge. The MID may change depending on the anchor used, the definition of ‘important change’ for that anchor, the type of anchor, the baseline values and the direction of change [14,18]. We used the change of MRS and BI as an anchor. Both scales have an acceptable degree of reliability in stroke [33]. The relationship was a linear positive correlation between the score changes for both indices with the categories of MRS and BI. We thus selected them as anchors. We found that stroke patients who reported global worsening had considerably larger score changes on the EQ-5D index than those reporting comparable global improvement in MRS anchored group (p = 0.032). On the other hand, stroke patients who reported global improvement had considerably larger score changes on the SF-6D index than those reporting comparable global worsening in BI anchored group (p = 0.045). In other words, the MID for the EQ-5D and SF-6D indices is likely to differ according to the direction of change or anchor used. We therefore displayed a range of MID values to account for this diversity.

A study reviewing eight longitudinal studies in eleven patient groups, using both, EQ-5D and SF-36, reported that the mean MID for the EQ-5D index was 0.074 (ranging from −0.011 to 0.140), and the mean MID for the SF-6D index was 0.041 (ranging from 0.011 to 0.097) [18]. Pickard et al. reported that MID estimate for the EQ-5D ranged from 0.10–0.14 in cancer patients based on performance status [7]. When calculating MID based on distribution in a previously published study, MID estimates ranged from 0.11–0.19 for the EQ-5D index and the corresponding estimates of the SF-6D index ranged from 0.03–0.08 [19].

Given that the range of the EQ-5D index (−0.171 to 1.0) was wider than that of the SF-6D index (0.296 to 1.00), the SD of the EQ-5D index was generally higher than that of the SF-6D index. The estimates of MID for the EQ-5D were approximately twice those of MID for the SF-6D index, except for the BI-anchored MID of SF-6D for patients with a minimally better state. Nevertheless, the MID estimates for each index seem to be equivalent, considering the range of index scores for each instrument.

This study also evaluated the responsiveness of the EQ-5D and SF-6D indices. Our findings suggest that both, the EQ-5D index and the SF-6D index were able to show changes of health state (i.e., both, improvement and deterioration) in stroke patients over the course of a 10-months period. The corresponding SRMs and ESs for EQ-5D of patients with a minimal changing state ranged from 0.27–0.50, and those for SF-6D of patients with a minimal changing state ranged from 0.27–1.11. Most of these values mainly indicated a small-to-medium effect based on Cohen’s criteria [32]. Findings of responsiveness of the EQ-5D and SF-6D in stroke in our study were consistent with the results of Pickard et al. [19]. However, EQ-5D and SF-6D was not equally responsive to change in other disease group such as cataract and heart failure patients [34,35] Researchers should be cautious the MID estimates and consider domains of preference-based measures may not detect the change according to disease characteristics.

There are several limitations of our study. The assessment on different time points was performed by different interviewers and we did not evaluate the inter-rater reliability in this study. However, there is evidence that the BI is highly reliable when recorded by nurses [36]. Thus, even though different interviewers assessed MRS and BI on the same person at different time points, the variability should be less due to the qualification and training course of the interviewers before survey. We arbitrarily defined ‘important change’ as the change of one grade on MRS and a change of 3–6 points in the BI score. A validation study would be required for the usage of both, MRS and BI as anchor. Sample size of BI anchored- minimally better group was small, therefore the estimated of the MID could be unstable. The MID estimates for SF-6D may be of limited value for the assessment in a Korean population, because we used an algorithm based on populations in the United Kingdom. However, MID estimates for EQ-5D using the Korean algorithm in our study were similar to the results of other researches using algorithms based on populations in the United Kingdom and the United States of America [7].

## Conclusion

The MIDs for these two utility measures slightly differed in their absolute magnitudes and by direction of change. Those MID estimates may be valuable for the interpretation of intervention effects related to HRQoL, particular in stroke patients and for the calculation of sample sizes for research studies.

## Abbreviations

BI: Barthel ADL index
HRQoL: Health-related quality of life
MID: Minimally Important Difference
MRS: Modified Rankin Scale
QALYs: Quality-Adjusted Life Years
SEM: Standard Error of Measurement
SF-36 v2: Short Form-36 version 2
